# A Bayesian decision support sequential model for severity of illness predictors and intensive care admissions in pneumonia

**DOI:** 10.1186/s12911-019-1015-5

**Published:** 2019-12-30

**Authors:** Amado Alejandro Baez, Laila Cochon, Jose Maria Nicolas

**Affiliations:** 1 University of Barcelona, Barcelona, Spain; 2 Universidad Nacional Pedro Henriquez Urena (UNPHU), Postgraduate Studies, Santo Domingo, Dominican Republic; 3 Medical College of Georgia, Department of Emergency Medicine, Augusta, GA USA

## Abstract

**Background:**

Community-acquired pneumonia (CAP) is one of the leading causes of morbidity and mortality in the USA. Our objective was to assess the predictive value on critical illness and disposition of a sequential Bayesian Model that integrates Lactate and procalcitonin (PCT) for pneumonia.

**Methods:**

Sensitivity and specificity of lactate and PCT attained from pooled meta-analysis data. Likelihood ratios calculated and inserted in Bayesian/ Fagan nomogram to calculate posttest probabilities. Bayesian Diagnostic Gains (BDG) were analyzed comparing pre and post-test probability. To assess the value of integrating both PCT and Lactate in Severity of Illness Prediction we built a model that combined CURB65 with PCT as the Pre-Test markers and later integrated the Lactate Likelihood Ratio Values to generate a combined CURB 65 + Procalcitonin + Lactate Sequential value.

**Results:**

The BDG model integrated a CUBR65 Scores combined with Procalcitonin (LR+ and LR-) for Pre-Test Probability Intermediate and High with Lactate Positive Likelihood Ratios. This generated for the PCT LR+ Post-test Probability (POSITIVE TEST) Posterior probability: 93% (95% CI [91,96%]) and Post Test Probability (NEGATIVE TEST) of: 17% (95% CI [15–20%]) for the Intermediate subgroup and 97% for the high risk sub-group POSITIVE TEST: Post-Test probability:97% (95% CI [95,98%]) NEGATIVE TEST: Post-test probability: 33% (95% CI [31,36%]) . ANOVA analysis for CURB 65 (alone) vs CURB 65 and PCT (LR+) vs CURB 65 and PCT (LR+) and Lactate showed a statistically significant difference (*P* value = 0.013).

**Conclusions:**

The sequential combination of CURB 65 plus PCT with Lactate yielded statistically significant results, demonstrating a greater predictive value for severity of illness thus ICU level care.

## Background

Community-acquired pneumonia (CAP) is one of the leading causes of death in the United States. The overall rate of CAP in adults is approximately 5.16 to 6.11 per 1000 persons per year [1, 2]. Risk factors include male gender, African American ethnicity, older age, and medical comorbidities [1]. In 2005, there were over 60,000 deaths due to pneumonia in the United States. CAP patients who require hospitalization experience higher mortality rates. According to data from the Centers for Medicare and Medicaid Services database, the estimated 30-day mortality rate of CAP patients requiring hospital admission in the United States is approximately 12% [2].

Patients who present to the emergency department with suspected CAP display a variety of symptoms including history of cough, dyspnea, pleuritic chest pain, acute functional or cognitive decline, fever, and tachycardia. Diagnosis of CAP is typically confirmed by chest radiography [1]. Emergency physicians must determine the severity of the patient’s illness and decide whether intensive care unit (ICU) level care if warranted. Clinical decision rules such as CURB65 risk score assist in the prediction of care needed, helping emergency physicians in making this determination.

Clinical decision rules use signs and symptoms along with imaging and laboratory test results to predict the probability of a patient having a specific condition [3, 4]. The CURB 65 Score is a widely used clinical decision rule to determine the severity of pneumonia and clinical estimation of management required [2]. The score ranges from 0 to 5, receiving one point each for the presence of **C**onfusion, Blood **U**rea Nitrogen (BUN) > 19 mg/dL, **R**espiratory rate ≥ 30 breaths per minute, systolic **B**lood pressure < 90mmHG or diastolic BP ≤ 60 mmHg, and age ≥ **65**, hence the acronym CURB-65. Each score has a corresponding estimated 30-day mortality risk percent and patient disposition suggestion. A score 0–1 advises outpatient care, while a score of 2 indicates patient discharge should be made at the physician’s discretion. A score of 3 or more advises inpatient admission and consideration for ICU admission with scores of 4 or 5 [5].

Serum biomarkers for infection are also used as predictors for sickness severity and level of care needed. Procalcitonin (PCT) is a 116 amino acid precursor of calcitonin, that during severe systemic inflammation, PCT is secreted in large quantities from many tissues. PCT serves as a specific marker for severe infections, in particular those caused by bacterial infection. Noninfectious inflammatory stimuli must be extremely severe in order to show PCT elevations. False-negative elevations of PCT are rare due to the fact PCT elevations are more sustained compared to other biomarkers and occur in neutropenic patients. PCT is detectable as soon as 2 to 4 h after a triggering event and peaks by 12 to 24 h. PCT is eliminated with a half-life of 24–35 h in the absence of a sustained triggering event. Higher levels of PCT are associated with more severe disease while declining levels represent resolution of illness [6]. In patients with CAP, monitoring of PCT may be useful as a predictor of treatment outcome [7]. Studies have also found PCT can also distinguish between gram-negative and gram-positive infection, as well as between different bacterial species and infection sites [8]. The use of PCT monitoring has led to significantly decreased median antibiotic exposure in patients with CAP [1].

Similarly, lactate is an organic compound produced by most tissues in the human body, most commonly from muscle. In anaerobic conditions, lactate is the end product of glycolysis. Lactate levels serve as a marker for illness severity and to gauge response to therapeutic interventions. Tissue hypoperfusion represents the most common cause of elevation. However there are many other contributing etiologies such as cardiac arrest, trauma, seizures, excessive muscle activity, regional ischemia, burns and smoke inhalation, diabetic ketoacidosis, thiamine deficiency, malignancy, liver dysfunction, inborn errors of metabolism, and medications [9]. Studies have shown monitoring of blood lactate levels in patients with severe pneumonia can serve as a prognosis indicator and to evaluate therapeutic management [10].

Bayesian statistics is a system for describing epistemological uncertainty using the mathematical language of probability, Bayesian statistics is a powerful “data recycling” tool of use in clinical decision making, and can be used to compare the diagnostic quality of different serum biomarkers, its methodology outputs the probability of an event based on criteria related to the specific event [4, 11–19]. Our group has developed a simple informatics method for interpreting diagnostic impact called “Bayesian Diagnostic Gains (BDG)”, where relative diagnostic gain (RDG) and absolute diagnostic gain (ADG) were calculated based on the differences deducted from pre- and posttest probabilities (ADG = post-test – pre-test) and (RDG = 100 × post-test – pre-test/Pre-test) [11–19]. This particular study is our first attempt at integrating BDGs in a sequential multi-item model.

The objective of this study was to assess the predictive value of sequential Bayesian decision model that integrates the CURB 65 Score, with procalcitonin (PCT) and Lactate, this to better elucidate intensive care unit (ICU) decision to admit in patients with pneumonia.

## Methods

Sensitivity and specificity of lactate and PCT were extracted from pooled meta-analysis data [7, 20]. Likelihood ratios were calculated using sensitivity and specificity to quantify the possibility of particular test results [7, 20]. (Table 1) The likelihood ratios were then inserted in a Bayesian model to calculate posttest probabilities.

**Table 1:** Sensitivity and Specificity of Serum Markers

	Sensitivity	Specificity	LR+	LR-
PCT	88.0%	81.0%	4.63	0.15
Lactate	72.7%	96.2%	19.0	0.28

^1^PCT: Procalcitonin

^1^LR+: Positive Likelihood Ratio

^1^LR-: Negative Likelihood Ratio

The results from the CURB 65 score were used as pretest probability alone and combined PCT likelihood ratios. To assess the value of integrating both Pro-Calcitonin and Lactate in Severity of Illness Prediction, we built a model that combined CURB65 with Pro-Calcitonin as the Pre-Test markers and later integrated the Lactate Likelihood Ratio Values to generate a combined CURB 65 + Procalcitonin + Lactate Sequential value integrated with Lactate in a Bayesian model to predict ICU admission.

The study population was risk stratified using point score ranges using CURB 65 for pneumonia severity. A score of 3 was considered intermediate risk of admission and scores 4–5 were high risk. Each subpopulation was attributed a risk percentage: 14% for intermediate risk and 27.8% for high risk. Procalcitonin was categorized into two groups: < 0.5 (negative), 0.5–2.0, and > 2.0 ng/ml (positive), a lactate level > 2 mmol/L was considered positive for the purposes of this study [7, 9]. The risk percentages were used as pre-test probability in the Bayesian/Fagan nomogram. Posttest probabilities were attained from the nomogram after inserting the CURB 65 scores (alone) or CURB 65 + PCT (LR+ and LR-) as pretest probability and likelihood ratios of each diagnostic test individually (Tables 2-3).

**Table 2:** PCT results for Positive Likelihood Ratios (LR+)

CURB 65 Score	Pretest	Post Test LR+	Absolute Gain	Relative Gain
Intermediate	14.0%	43.0%	29.0%	207.1%
High	27.8%	64.0%	36.3%	130.6%

^1^PCT: Procalcitonin

^1^LR+: Positive Likelihood Ratio

^1^LR-: Negative Likelihood Ratio

**Table 3:** PCT results for Negative Likelihood Ratios (LR-)

CURB 65 Score	Pretest	Post Test	Absolute Gain	Relative Gain
Intermediate	14.0%	2.0%	− 12.0%	85.7%
High	27.8%	5.0%	−22.8%	82.0%

^1^PCT: Procalcitonin

^1^LR+: Positive Likelihood Ratio

^1^LR-: Negative Likelihood Ratio

To quantify diagnostic impact, we developed a framework called “Bayesian Diagnostic Gains (BDG)”, where relative (RDG) and Absolute (ADG) diagnostic gains were calculated using differences between CURB 65 pretest results and post test probabilities. Absolute gain was defined as the difference between pretest and post test probability (ADG = Post- Pre). Relative gain was the percentage of absolute gain in relation to pretest probability (RDG = ADG/Pre × 100).

“Number Needed to” metrics hold a more intuitive appeal for clinicians than standard diagnostic accuracy measures and these tools are being used for correctly treating, diagnose or predict disease in certain populations [15]. The Number Needed to Treat (NNT) is the number of patients you need to treat to prevent one additional bad outcome. The NNT is the inverse of the absolute risk reduction (ARR). The ARR is the absolute difference in the rates of events between a given activity or treatment relative to a control activity or treatment, i.e. control event rate (CER) minus the experimental event rate (EER), or ARR = CER - EER. The NNTs are always rounded up to the nearest whole number and accompanied as standard by the 95% confidence interval. Example: if a drug reduces the risk of a bad outcome from 50 to 40%, the ARR = 0.5–0.4 = 0.1. Therefore, the NNT = 1/ARR = 10. The ideal NNT would be 1 - ie all patients treated will benefit [14].

On the basis of this concept, we used the ADG to create a formula for the Number Needed to Diagnose (NND) and called it Bayesian Number Needed to Diagnose (B-NND). For this tool we took the statistical basis of the formula used for the NNT, using ADG as a substitute for ARR. Our formula is as follows: NND = 1/ADG.

ANOVA statistics were used to evaluate the strength of association with a *p* value set at 0.05 (Figs. 4 and 5). R version 3.5.2 (2018-12-20) -- “Eggshell Igloo”. Copyright (C) 2018 The R Foundation for Statistical Computing.

Platform: i386-w64-mingw32/i386 (32-bit) was used for analysis and TreeAge pro 2019 for decision tree schematization and analysis. This was an Institutional Review Board Exempt study.

## Results

Pooled diagnostic quality data obtained from meta-analysis (Table-1) reflected a sensitivity for PCT as 88% (95% confidence interval (CI): 80–93%) and specificity of 81% (95% CI: 67–90%) [17]. A meta-analysis for lactate reported a sensitivity of 72.7% (95% CI: 43.4, 90.2%) and specificity of 96.2% (95% CI: 90.6, 98.5%) [18]. This resulted in Likelihood Ratios (LR) for PCT were LR+ 4.63 and LR- 0.15. LR for lactate resulted as LR+ 19.0 and LR- 0.28.

Inserting CURB 65 risk score as pretest probability and positive LR in Bayes Sensitivity and Specificity Sequential tree analysis generated a posttest probability for PCT and lactate (Tables 2 and 3). Results for PCT intermediate risk yielded a post test probability of 43.0% (ADG = 29.0%, RDG = 207.1%). PCT high risk post test probability was 64.0% (ADG = 36.3%, RDG = 130.6%). Using Lactate alone as diagnostic marker on a Bayes CURB 65 Model (Tables 4 and 5) yielded a post-test probability for intermediate risk was 76% (ADG = 62%, RDG = 442.9%). Lactate high risk posttest probability was 88% (ADG = 60.2%, RDG = 216.6%).

**Table 4:** Lactate results for Positive Likelihood Ratios (LR+)

CURB 65 Score	Pretest	Post Test LR+	Absolute Gain	Relative Gain
Intermediate	14.0%	76.0%	62.0%	442.9%
High	27.8%	88.0%	60.2%	216.6%

^1^LR+: Positive Likelihood Ratio

^1^LR-: Negative Likelihood Ratio

**Table 5:** Lactate results for Negative Likelihood Ratios (LR-)

CURB 65 Score	Pretest	Post Test	Absolute Gain	Relative Gain
Intermediate	14.0%	4.0%	−10.0%	71.4%
High	27.8%	10.0%	−17.8%	64.0%

^1^LR+: Positive Likelihood Ratio

^1^LR-: Negative Likelihood Ratio

**Fig. 1:**
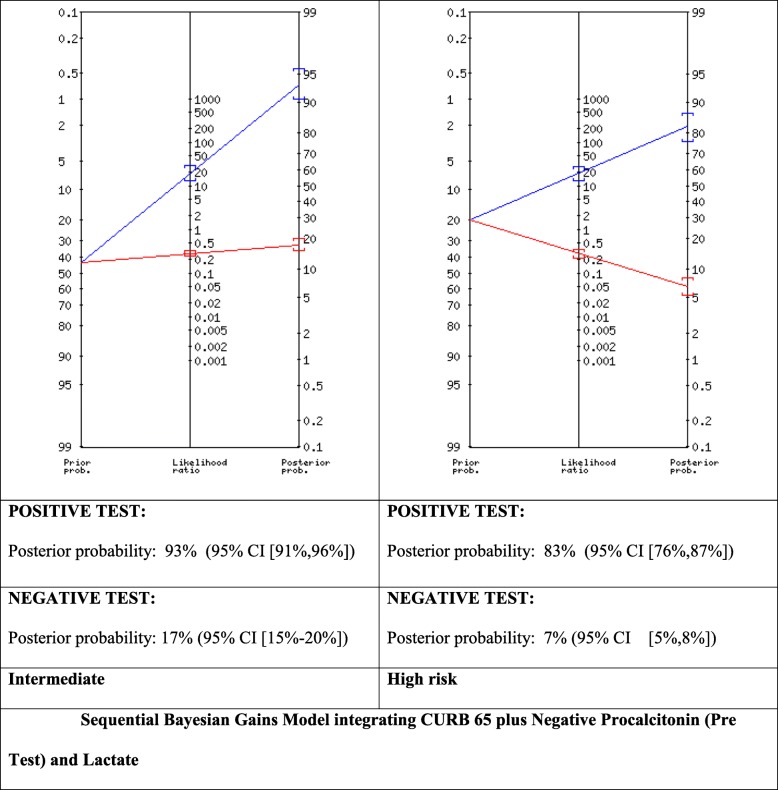
Sequential Bayesian Gains Model integrating CURB 65 plus Negative Procalcitonin (Pre Test) and Lactate

**Fig. 2:**
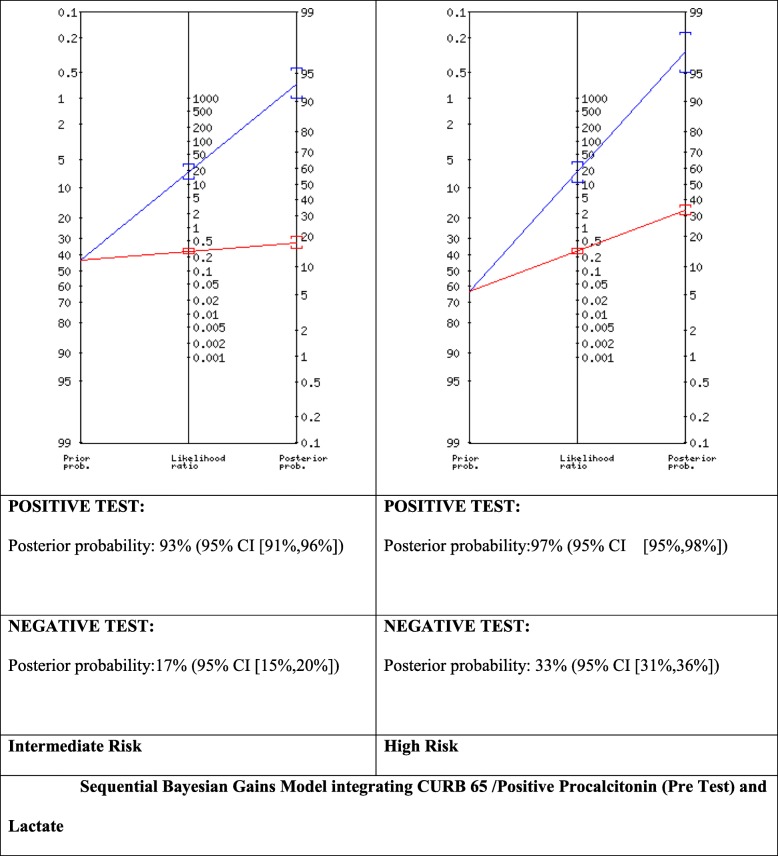
Sequential Bayesian Gains Model integrating CURB 65 /Positive Procalcitonin (Pre Test) and Lactate

We proceeded to build and experimental Bayesian Gains Sequential model that integrated a CUBR65 Intermediate (Int) (14% prevalence) Scores combined with Procalcitonin (both LR+ and LR-) for Pre-Test Probability Intermediate (43%) and High (67%) with Lactate Positive Likelihood Ratios (Fig. 1, Fig. 2). This generated for the PCT LR+ (Fig. 1) Post-test Probability (POSITIVE TEST) Posterior probability: 93% (95% CI [91,96%]) and Post Test Probability (NEGATIVE TEST) of: 17% (95% CI [15–20%]) for the Intermediate subgroup and 97% for the high risk sub-group POSITIVE TEST: Post-Test probability:97% (95% CI [95,98%]) NEGATIVE TEST: Post-test probability: 33% (95% CI [31,36%]) . Whereas for CURB65 (Intermediate) integrated with PCT LR- (Fig. 2) for Intermediate and High Risk subgroups found for the intermediate risk subgroup a POSITIVE TEST: Posterior probability: 93% (95% CI [91,96%]) NEGATIVE TEST: Posterior probability: 17% (95% CI [15–20%]) POSITIVE TEST: Posterior probability: 83% (95% CI [76,87%]) and NEGATIVE TEST: Posterior probability:7% (95% CI [5,8%]).

When we applied the formula for B-NND we obtained the following results using CURB 65 Intermediate Score (14%). When calculating B-NND for CURB 65-I integrated with PCT LR + (ADG 29% yielded a B-NND of 3.45, whereas hen combining PCT (+) with Lactate (+) ADG 79.0%) for a B-NND of 1.27 and finally, CURB-65 Intermediate and only Lactate (+) resulted in an ADG of 62% for a B-NND of 1.61 (Table 6).

**Table 6:** Bayesian Number Needed to Diagnose

Pre-test Probability	Post- test (LR+)	ADG	B-NND (rounded)
CURB 65 (14.0%)	PCT (alone) 43.0%	29.0%	3.45 (3)
CURB 65 (14.0%)	PCT and Lactate 93.0%	79.0%	1.27 (1)
CURB 65 (14.0%)	Lactate (alone) 76.0%	62.0%	1.61 (2)

^1^LR+: Positive Likelihood Ratio

^1^ADG: Absolute Diagnosis Gain

^1^B-NND: Bayesian Number Needed to Diagnose

**Fig. 3:**
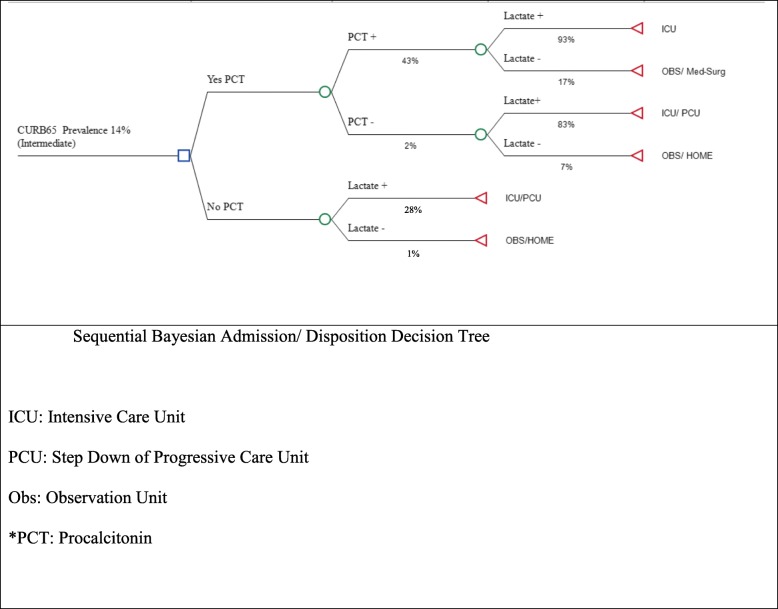
Sequential Bayesian Admission/ Disposition Decision Tree. ICU: Intensive Care Unit. PCU: Step Down of Progressive Care Unit. Obs: Observation Unit. *PCT: Procalcitonin

ANOVA analysis for CURB 65 (alone) vs CURB 65 and PCT (LR+) vs CURB 65 and PCT (LR+) and Lactate showed a statistically significant difference (*P* value = 0.013), with a f-ratio value of 25.56 (Fig. 3). Whereas no statistical significance was found in the Negative Likelihood Ratio sequential model (Fig. 2).

A simple admission decision tree was developed for the “Intermediate Risk Sub Group” based on this Sequential Bayesian Diagnostic Gains Model (Fig. 3), where an “intermediate CURB 65” integrated with a positive PCT and Positive Lactate should warrant ICU admission, and other integrations of procalcitonin and lactate in independent and sequential iterations generate specific disposition decisions based on post-test probability assessments.

## Discussion

According to the CURB 65 score, high risk patients are those that should be considered for an admission to the ICU. The Bayesian statistical model demonstrated a superior diagnostic gains in predicting ICU admissions with the independent integration of lactate compared to Procalcitonin. Absolute diagnostic gain was greater for both lactate and PCT in the intermediate risk category, showing a more important gain in the lactate subgroup. However, in high risk patients relative gain and absolute gains were not a meaningful, but still favoring Lactate.

**Fig. 4:**
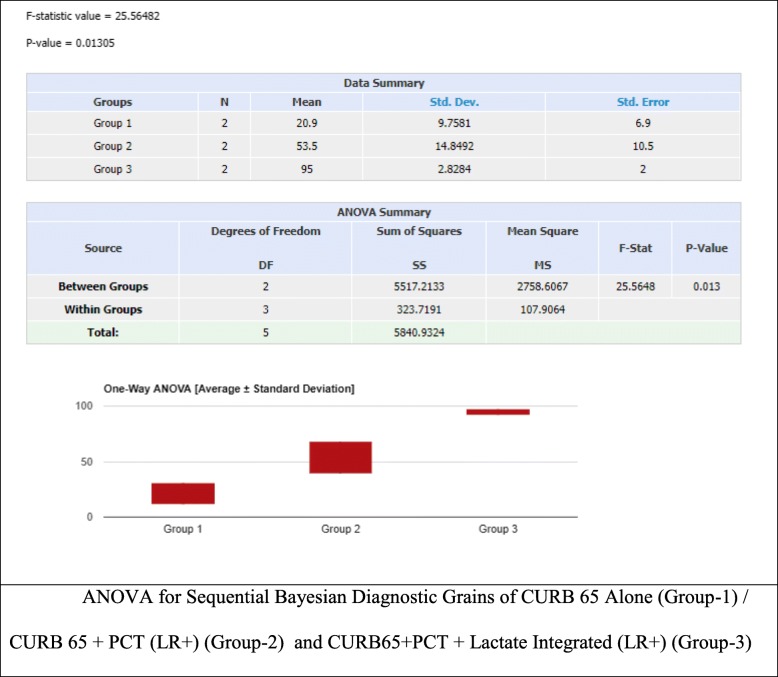
ANOVA for Sequential Bayesian Diagnostic Grains of CURB 65 Alone (Group-1) / CURB 65 + PCT (LR+) (Group-2) and CURB65 + PCT + Lactate Integrated (LR+) (Group-3)

The sequential integration of Pro-Calcitonin (LR+) plus CURB 65 combined with Lactate demonstrated a high post-test probability, almost similar in intermediate (93%) and high pretest probability (97%) showing an absolute difference of only 4%, and supporting its use more meaningfully in the intermediate pre-test subgroups, this demonstrated a statistically significant value (Fig. 4). Confirming that patient-centered clinical decision making should be able to integrate several clinical items in an effort to adequately predict severity of illness and eventual ICU resource utilization.

**Fig. 5:**
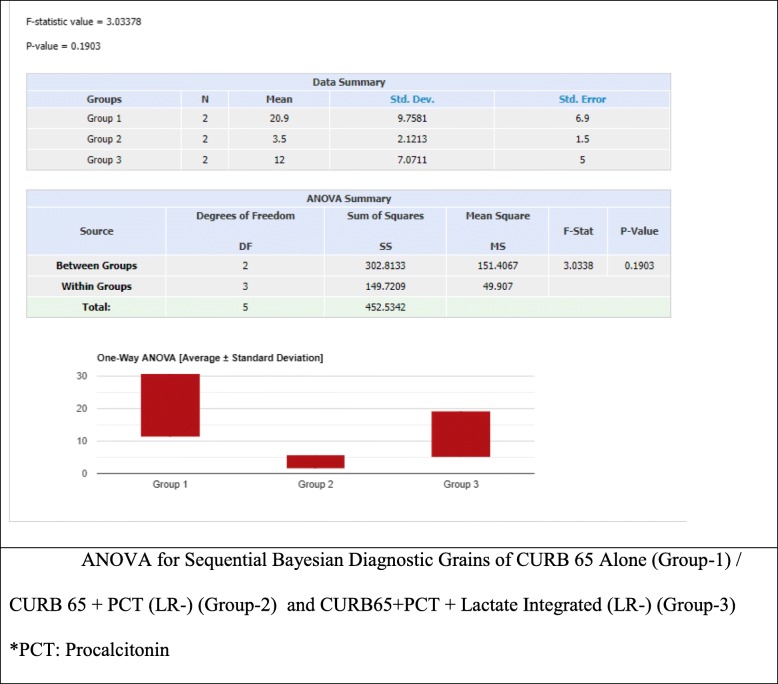
ANOVA for Sequential Bayesian Diagnostic Grains of CURB 65 Alone (Group-1) / CURB 65 + PCT (LR-) (Group-2) and CURB65 + PCT + Lactate Integrated (LR-) (Group-3). *PCT: Procalcitonin

These results are important in that they suggest Lactate integrated with the CURB 65/ PCT (LR+) have a greater predictive value for ICU admissions in patients with pneumonia. Whereas, no statistically significant sequential improvement was found in the LR (−) predictive model (Fig. 5).

Our Decision Support Tree (Fig. 3) integrates these findings, suggesting an integrated approach to admission decision making that contemplates CURB65, PCT and Lactate in admission decision making.

The cost effectiveness of this decision process needs to be further elucidated, as it is an enticing concept for health economics. From a research stand point, by mathematically integrating results from strong but independent research studies and performing a “data recycling study” one can develop hypothesis generating and decision support tools, without costly investments in independent studies, our group has produced and validated this model in multiple other studies, [4, 15–19] thus creating a new reference instrument for evaluating integrating independent data results and as a hypothesis generating tool. Clinically this process can also justify a positive health economics impact. In a 2013 study performed by Smith et al. found PCT protocols cost $10–$54 more per patient than usual care in CAP patients [21]. Lactate level costs vary from hospital to hospital, with an average test of costs $78 [22]. The average cost of Intensive Care Unit stay in the United States ranges around >$4000/ day, thus by properly integrating the combination of CURB65, Procalcitonin and Lactate systems can me reliably predict need for ICU stay and potentially save thousands of dollars on individual not recommended ICU admissions, and very likely saving millions of dollars on unnecessary ICU admissions at a more macro level. A more comprehensive value-based and cost effective analysis will be performed in a future study that also prospectively contemplates disposition making and decision support. Furthermore, future studies should take into consideration the cost of measuring PCT or lactate when choosing which test to use in guiding patient care and decision making. Currently our group is generating a validation study that integrates real (retrospective) patient data in assessing decision making effectiveness and cost.

Limitations of this study include the need for prospective validation of this method given that our study represents a mathematical estimation. Our study is based on meta-analysis data that needs to be further validated. Other limitations include but are not limited to individual systems issues that influence admission decisions which would limit our study’s generalizability. The point in time at which the blood level of these biomarkers are obtained is also a limitation due to the wide variation of values at different points in the disease process.

## Conclusion

Bayesian statistical model demonstrated a superior independent diagnostic gain in predicting ICU admissions with the integration of lactate to the CURB 65 risk score. The sequential combination of CURB 65 plus Procalcitonin with Lactate yielded statistically significant results, showing that the integration of Lactate with the CURB 65 Risk Score plus Procalcitonin had a greater predictive value for severity of illness thus ICU level care, thus suggesting that decision support tools be able to combine these various clinical items in a final decision pathway for the prediction of ICU admissions in patients with pneumonia.

## Data Availability

Data sharing is not applicable to this article as no datasets were generated or analysed during the current study.Not applicable.

## Abbreviations

ACDC: Acute Care Diagnostics Collaboration
ADG: Absolute Diagnostic Gains
ANOVA: Analysis of Variance
BDG: Bayesian Diagnostic Gains
B-NND: Bayesian Number Needed to Diagnose
CAP: Community Acquired Pneumonia
PCT: Procalcitonin

## References

[1] Kaysin A, Viera AJ (2016). Community-acquired pneumonia in adults: diagnosis and management. Am Fam Physician.

[2] Lim WS, van der Eerden MM, Laing R, Boersma WG, Karalus N, Town GI (2003). Defining community acquired pneumonia severity on presentation to hospital: an international derivation and validation study. Thorax.

[3] Reilly BM, Evans AT (2006). Translating clinical research into clinical practice: impact of using prediction rules to make decisions. Ann Intern Med.

[4] Cochon L, McIntyre K, Nicolás JM, Baez AA (2017). Incremental diagnostic quality gain of CTA over V/Q scan in the assessment of pulmonary embolism by means of a Wells score Bayesian model: results from the ACDC collaboration. Emerg Radiol.

[5] CURB-65 Score for Pneumonia Severity - MDCalc [Internet]. Available from: https://www.mdcalc.com/curb-65-score-pneumonia-severity. [cited 2018 Dec 1]

[6] PCT - Clinical: Procalcitonin, Serum [Internet]. Available from: https://www.mayomedicallaboratories.com/test-catalog/Clinical+and+Interpretive/83169. [cited 2018 Dec 1]

[7] Serum procalcitonin and C-reactive protein levels as markers of bacterial infection: a systematic review and meta-analysis. - PubMed - NCBI [Internet]. Available from: https://www.ncbi.nlm.nih.gov/pubmed/15307030. [cited 2018 Dec 1]

[8] Yan ST, Sun LC, Jia HB, Gao W, Yang JP, Zhang GQ (2017). Procalcitonin levels in bloodstream infections caused by different sources and species of bacteria. Am J Emerg Med.

[9] Andersen LW, Mackenhauer J, Roberts JC, Berg KM, Cocchi MN, Donnino MW (2013). Etiology and therapeutic approach to elevated lactate levels. Mayo Clin Proc.

[10] LIU W, PENG L, HUA S (2015). Clinical significance of dynamic monitoring of blood lactic acid, oxygenation index and C-reactive protein levels in patients with severe pneumonia. Exp Ther Med.

[11] Medow MA, Lucey CR (2011). A qualitative approach to Bayes’ theorem. Evid Based Med.

[12] Bonabeau E (2003). Don’t trust your gut. Harv Bus Rev.

[13] Schriger D, Elder J, Cooper R (2017). Structured clinical decision aids are seldom compared with subjective physician judgment, and are seldom superior. Ann Emerg Med.

[14] Penaloza A, Verschuren F, Meyer G, Quentin-Georget S, Soulie C, Thys F (2013). Comparison of the unstructured clinician gestalt, the wells score, and the revised Geneva score to estimate pretest probability for suspected pulmonary embolism. Ann Emerg Med.

[15] Baez AA, Cochon L (2018). The acute care diagnostics collaboration: performance assessment of contrast-enhanced ultrasound compared to abdominal computed tomography and conventional ultrasound in an emergency trauma score bayesian clinical decision scheme. Int J Crit Illn Inj Sci.

[16] Farook N, Cochon L, Bode AD, Langer BP, Baez AA (2018). HEART score and stress test emergency department Bayesian decision scheme: results from the acute care diagnostic collaboration. J Emerg Med.

[17] Cochon L, Smith J, Baez AA (2017). Bayesian comparative assessment of diagnostic accuracy of low-dose CT scan and ultrasonography in the diagnosis of urolithiasis after the application of the STONE score. Emerg Radiol.

[18] Baez AA, Cochon L (2017). Improved rule-out diagnostic gain with a combined aortic dissection detection risk score and D-dimer Bayesian decision support scheme. J Crit Care.

[19] Baez AA, Cochon L (2016). Acute care diagnostics collaboration: assessment of a Bayesian clinical decision model integrating the Prehospital Sepsis score and point-of-care lactate. Am J Emerg Med.

[20] Zhang Z, Xu X (2014). Lactate clearance is a useful biomarker for the prediction of all-cause mortality in critically ill patients: a systematic review and meta-analysis*. Crit Care Med.

[21] Smith KJ, Wateska A, Nowalk MP, Raymund M, Lee BY, Zimmerman RK (2013). Cost-effectiveness of procalcitonin-guided antibiotic use in community acquired pneumonia. J Gen Intern Med.

[22] main-campus-hospital-patient-price-list.pdf [Internet]. Available from: https://my.clevelandclinic.org/-/scassets/files/org/locations/price-lists/main-campus-hospital-patient-price-list.ashx. [cited 2018 Dec 1]

